# Study on the Enrichment of the Main Active Components in *Rhodococcus opacus* PD630 Cell-Free Supernatant for the Degradation of Aflatoxin B1, the Degradation Products, and the Underlying Mechanisms

**DOI:** 10.3390/foods14213772

**Published:** 2025-11-03

**Authors:** Aiyuan Zhang, Xuewu Zhang, Jiguo Yang

**Affiliations:** 1 College of Food Science and Engineering, South China University of Technology, Guangzhou 510641, China; zhangaiyuan1110@163.com (A.Z.);; 2 South China Institute of Collaborative Innovation, Dongguan 523808, China

**Keywords:** AFB1, *Rhodococcus opacus* PD630, cell-free supernatant, ethanol precipitation, metabolites and pathways

## Abstract

Due to the high toxicity and widespread distribution of aflatoxin B1 (AFB1), there is significant interest in efficient, safe, and environmentally friendly microbial degradation methods. *Rhodococcus opacus* PD630 cell-free supernatant (RCFS) shows excellent activity in degrading AFB1, but its active components and mechanisms remain unclear. We assessed the feasibility of ethanol precipitation to enrich active components in RCFS and characterized the ethanol precipitate (RCFSC-EP). Metabolomics and proteomics were used to elucidate the active components, mechanisms, and products of AFB1 degradation by RCFS. The results indicate that ethanol precipitation enriches over 80% of the active components for AFB1 degradation in RCFS. RCFSC-EP exhibits excellent heat resistance, and inhibitors like EDTA-2Na and proteinase K significantly inhibit its activity. Multi-omics analysis suggests that active components in RCFS metabolize AFB1 into six products through four potential pathways, three of which withstand 135 °C for 20 min. The AFB1-degrading activity of RCFS is an intrinsic, constitutive trait of *R. opacus* PD630 during normal growth. The active components are diverse proteins or enzymes, including glutathione S-transferases, aldo/keto reductase, peroxidases, and carbonyl reductases. This study enriches and reveals the active components, pathways, and products of AFB1 degradation by RCFS, providing a basis for developing RCFS as a biological agent for AFB1 degradation.

## 1. Introduction

Mycotoxins are toxic secondary metabolites synthesized by certain toxigenic strains of *Aspergillus*, *Fusarium*, *Alternaria*, and *Penicillium* during their growth [1], and are among the most hazardous chemical contaminants in food and feed [2]. They exhibit carcinogenic, mutagenic, teratogenic, and hepatotoxic effects [3,4]. Cereals are the primary targets of toxigenic strains within *Aspergillus*, *Fusarium*, and *Penicillium* [5]. The Food and Agriculture Organization (FAO) estimates that approximately 25% of cereals are contaminated by mycotoxins annually [2], and animals can also be exposed through consumption of contaminated feed [6], posing significant health risks to both humans and livestock.

Aflatoxins (AFs) are primarily produced by toxigenic strains of *Aspergillus flavus* and *Aspergillus parasiticus* in foods such as peanuts, maize, rice, nuts, cocoa beans, dried fruits, spices, and crude vegetable oils [6]. Among the approximately twenty known AFs, AFB1 is the most prevalent and exhibits the highest toxicity [7]. The liver serves as the principal target organ; AFB1 induces hepatitis and hepatocellular carcinoma and can be lethal, acting as a potent human carcinogen [8,9]. After ingestion, AFB1 is converted to AFB1-exo-8,9-epoxide (AFBO) by cytochrome P450 enzymes. AFBO binds to the N7 position of guanine in DNA, and the AF-N7-guanine adduct induces GC → TC transversion mutations [10,11]. Due to its high toxicity and widespread occurrence, AFB1 limits in various foods have been set by different countries and regions; for instance, the European Commission stipulates a maximum level of 2 µg/kg AFB1 in any product intended for direct human consumption.

Extensive research has been conducted on AFB1 degradation in food using physical, chemical, and biological approaches. Physical and chemical treatments are partially effective, but their high cost, accompanied by losses in nutritional value and sensory quality, limits practical application; their safety and efficacy require further evaluation [12]. In contrast, microbial degradation of AFB1 is attractive because it is efficient, safe, and eco-friendly. To date, numerous microorganisms capable of AFB1 detoxification have been identified. Among bacteria, strains of *Bacillus*, *Actinomycetes*, and *γ-Proteobacteria* degrade AFB1 enzymatically, with active principles located intracellularly or as extracellular enzymes. Non-toxigenic *Aspergillus* spp. and certain *Basidiomycota* fungi also degrade AFB1 enzymatically, with active components distributed intracellularly, in cell-free culture supernatants, or in culture filtrates [13].

As a member of the *Actinomycetes*, *Rhodococcus* is a Gram-positive, strictly aerobic genus widely distributed in soil, marine, and polar habitats. It possesses a large genome, megaplasmids, and redundant, versatile metabolic pathways, making it industrially attractive for biotransformation and biodegradation [14]. Ludmila et al. [14] reported that *R. erythropolis* secretes various enzymes involved in aromatic compound degradation, including ring-cleavage dioxygenases, biphenyl dioxygenase, dihydrodiol dehydrogenase, and hydrolases, with all central aromatic degradation pathways terminating in the citric acid cycle. AFB1, a polyaromatic mycotoxin, could therefore be channeled through analogous pathways. Alberts [15] first demonstrated that extracellular extracts of *R. erythropolis* enzymatically degrade AFB1, yielding non-mutagenic products in the Ames test. Krifation et al. [16] screened *Rhodococcus* strains for AFB1 degradation; fourteen species—including *R. pyridinivorans*, *R. erythropolis*, *R. rhodochrous*, and *R. globerulus*—completely detoxified AFB1, yielding metabolites devoid of genotoxicity. Anita et al. [17] further showed that intracellular enzyme preparations from *R. erythropolis* and *R. rhodochrous* degraded ≥80% of AFB1 within 6 h in a stable enzymatic process; the resultant products were non-genotoxic. Nevertheless, the precise active sites, product structures, and mechanistic pathways underlying AFB1 degradation by *Rhodococcus* remain to be elucidated.

Lapalikar et al. [18] identified and characterized the deazaflavin cofactor F_420_-H_2_-dependent FDR-A enzyme from *R. erythropolis* PR4, which catalyzes the reduction in the α,β-unsaturated ester moiety of AFB1. This reduction triggers spontaneous hydrolysis and detoxification, converting AFB1 into smaller compounds. The authors further postulated that once the (furan) coumarin system is reduced by FDR-As, the resulting intermediates could be funneled through the catechol degradation pathway to supply substrates for the citric acid cycle. Nevertheless, a comprehensive understanding of AFB1 degradation by *Rhodococcus* spp. still requires additional studies to elucidate the complete spectrum of degradation products and to identify the specific enzymes or enzymatic systems responsible for the biotransformation.

This study centers on the degradation of AFB1 by *R. opacus* PD630 cell-free supernatant (RCFS). Ethanol precipitation will enrich active constituents (RCFSC-EP), whose properties and process feasibility will be assessed. Metabolomic and proteomic analyses will map degradation products, elucidate mechanisms, and identify key proteases, laying the groundwork for RCFS-based AFB1-detoxifying formulations.

## 2. Materials and Methods

### 2.1. Materials and Equipment

The experimental strain *R. opacus* PD630 (DSMZ 44193) was obtained from the China General Microbiological Culture Collection Center (CGMCC). Nutrient Broth was purchased from Guangdong Huankai Microbial Sci. & Tech. Co., Ltd. (Guangzhou, China). Ultrapure water was prepared with a Milli-Q system (Millipore, Bedford, MA, USA). The reagents and equipment used in this study are detailed in Appendix A.

### 2.2. Preparation of R. opacus PD630 Seed Culture and Cell-Free Supernatant

An aliquot of log-phase *R. opacus* PD630 preserved in 25% (*v*/*v*) glycerol at −80 °C was inoculated into Nutrient Broth (NB) and incubated at 30 °C, 160 rpm, until OD_660nm_ = 1.0 to obtain the seed culture. This seed culture was then transferred into fresh NB medium at 2% (*v*/*v*) inoculum (≈10^7^ CFU/mL) and cultivated at 30 °C, 160 rpm, for 36 h. The cells were removed by centrifugation at 8000 rpm for 20 min, and the resulting supernatant was immediately filtered through a 0.22 µm membrane to yield the *R. opacus* PD630 cell-free supernatant (RCFS) used for subsequent experiments.

### 2.3. HPLC Determination of AFB1

Following Liu et al. [19] with minor modifications, a 40 µg/mL AFB1 stock solution was prepared by dissolving 1 mg of AFB1 reference standard in 25 mL HPLC-grade acetonitrile and stored in the dark at 4 °C. For the assay, 9.8 mL of RCFS (prepared in Section 2.2) was combined with 200 µL of the 40 µg/mL AFB1 stock to yield an initial AFB1 concentration of 800 µg/L. After thorough mixing, the reaction mixture was incubated at 30 °C, 160 rpm, in the dark for 24 h. The reaction solution was extracted three times with an equal volume of dichloromethane; the organic layers were pooled. The combined dichloromethane extract was evaporated to dryness under a gentle nitrogen stream, and the residue was reconstituted in 1 mL HPLC-grade acetonitrile. The reconstituted solution was filtered through a 0.22 µm organic membrane prior to HPLC analysis. Sterile NB medium supernatant subjected to the same treatment served as the negative control throughout. HPLC analysis was performed on a Shimadzu LC-20AD system (Shimadzu, Kyoto, Japan) equipped with a Waters C18 column (5 µm, 4.6 × 150 mm). The mobile phase consisted of acetonitrile/water (40:60, *v*/*v*) at a flow rate of 1.0 mL/min; column temperature was maintained at 30 °C. Injection volume was 20 µL, and detection was carried out with a UV detector at 365 nm.

The AFB1 degradation rate was calculated as follows:

AFB1 degradation (%) = [1 − (peak area of AFB1 in treatment/peak area of AFB1 in control)] × 100%(1)

### 2.4. Enrichment of AFB1-Degrading Active Principles from RCFS by Ethanol Precipitation

Active components in RCFS were enriched by absolute ethanol precipitation following Li et al. [20] with minor modifications. RCFS (1L) was first concentrated 10-fold at 55 °C under reduced pressure in a rotary evaporator; the retentate was reconstituted to the original volume to evaluate the effect of vacuum concentration alone (hereafter RCFSC). The concentrate was mixed with 4 volumes of absolute ethanol (final 80%, *v*/*v*) and held at −20 °C overnight. After centrifugation (8000 rpm, 20 min), the pellet was dissolved in sterile ultrapure water to the original volume (designated below as RCFSC-EP), whereas the supernatant was rotary-evaporated to remove ethanol and reconstituted to the original volume with sterile ultrapure water (designated below as RCFSC-ES). Sterile NB medium supernatant subjected to the same treatment served as the negative control. Protein contents of RCFS and RCFSC-EP were determined in parallel using the Coomassie Brilliant Blue G-250 assay. Each fraction was incubated with AFB1 (800 µg/L) at 30 °C, 160 rpm, in the dark for 24 h according to Section 2.3, and residual AFB1 was quantified by HPLC to evaluate the feasibility of ethanol precipitation for enriching AFB1-degrading activity.

### 2.5. Effect of Temperature on AFB1-Degrading Activity of RCFSC-EP

To evaluate the thermal stability of the AFB_1_-degrading principle(s) in RCFSC-EP, aliquots prepared in Section 2.4 were subjected to 20 min heat treatments at 80 °C, 100 °C, 120 °C, and 135 °C in an autoclave, following the protocol of Ref. [21] with minor modifications. Sterile NB supernatant subjected to the same temperature treatments served as the negative control. Each heat-treated sample was incubated with AFB1 (initial concentration 800 µg/L) at 30 °C, 160 rpm, in darkness for 24 h as described in Section 2.3. Residual AFB1 was extracted and quantified by HPLC (Section 2.3) to assess the thermotolerance of the active component(s) responsible for AFB1 degradation.

### 2.6. Effects of Proteinase K and EDTA-2Na on the AFB1-Degrading Activity of RCFSC-EP

To investigate the influence of EDTA-2Na and proteinase K, aliquots of RCFSC-EP were incubated with 10 mmol/L of EDTA-2Na or 1 mg/mL of proteinase K at 55 °C for 1 h in the dark. After inhibitor treatment, each sample was mixed with AFB1 (initial concentration 800 µg/L) and incubated at 30 °C, 160 rpm, in darkness for 24 h according to Section 2.3. The same inhibitor assays were simultaneously performed on RCFSC-EP that had been pretreated at 135 °C for 20 min (designated RCFSC-EP-H) to evaluate whether heat-stable activity was similarly affected. Sterile NB medium supernatant subjected to the same treatment served as the negative control, following the protocols described in Refs. [22,23]. Residual AFB1 was extracted and quantified by HPLC as detailed in Section 2.3.

### 2.7. Influence of Substrate Concentration on AFB1-Degrading Activity of RCFSC-EP

To evaluate the effect of substrate concentration on the AFB1-degrading activity of RCFSC-EP, the initial AFB1 concentration was set at 400, 800, 1200, and 1600 µg/L according to Ref. [24]. Each reaction mixture was incubated at 30 °C, 160 rpm, for 24 h, as described in Section 2.3. The persistence of the active principle(s) was further assessed by a modified repeated-substrate supplementation protocol adapted from He et al. [25]: 8 µg AFB1 was spiked into the 10 mL reaction system every 24 h until a cumulative AFB1 load of 4000 µg/L was reached; the total incubation time was 120 h under the same conditions. Parallel assays were conducted with heat-treated RCFSC-EP (135 °C, 20 min; designated RCFSC-EP-H) to determine whether thermostable activity was similarly influenced by substrate level. Sterile NB medium supernatant subjected to the same treatment served as the negative control. Residual AFB1 was extracted and quantified by HPLC as detailed in Section 2.3.

### 2.8. Non-Targeted Metabolomic Analysis of AFB1 Degradation Products by RCFS

#### 2.8.1. Sample Preparation for Metabolomic Profiling of AFB1 Degradation by RCFS

RCFS was prepared as described in Section 2.2, spiked with AFB1 standard to 800 µg/L, and incubated at 30 °C, 160 rpm, in the dark for 24 h. The mixture was immediately frozen at −80 °C and lyophilized, yielding sample RCFS-AFB1 for product analysis. A parallel set was prepared using RCFS pre-heated at 135 °C for 20 min (RCFS-H-AFB1) to identify products generated by heat-stable active components. RCFS without AFB1 served as the blank control (RCFS-CK). Three replicate samples were set up for experimental testing.

#### 2.8.2. Metabolomic Sample Pretreatment for RCFS

An appropriate amount of the lyophilized sample was weighed into a 2 mL tube, mixed with 600 µL of methanol containing 2-chloro-L-phenylalanine (4 ppm), and vortexed for 30 s. After adding a steel bead, the mixture was homogenized at 55 Hz for 60 s, sonicated at room temperature for 15 min, and centrifuged at 12,000 rpm, 4 °C, for 10 min. The supernatant was immediately filtered through a 0.22 µm membrane for LC-MS analysis.

#### 2.8.3. LC-MS Analysis of RCFS Non-Targeted Metabolomics

Chromatographic conditions: An ultra-high-performance liquid chromatography (UHPLC) system (Thermo Vanquish, Thermo Fisher Scientific, Waltham, MA, USA) was used, equipped with an ACQUITY UPLC^®^ HSS T3 column (2.1 × 100 mm, 1.8 µm, Waters, Milford, MA, USA). The flow rate was 0.3 mL/min, column temperature was 40 °C, and injection volume was 2 μL. Positive-ion mode: Mobile phases: 0.1% formic acid in acetonitrile (B2) and 0.1% formic acid in water (A2). Gradient: (a) 0–1 min, 10% B2; (b) 1–5 min, 10–98% B2; (c) 5–6.5 min, 98% B2; (d) 6.5–6.6 min, 98–10% B2; (e) 6.6–8 min, 10% B2. Negative-ion mode: Mobile phases: acetonitrile (B3) and 5 mM ammonium formate in water (A3). Gradient: (a) 0–1 min, 10% B3; (b) 1–5 min, 10–98% B3; (c) 5–6.5 min, 98% B3; (d) 6.5–6.6 min, 98–10% B3; (e) 6.6–8 min, 10% B3 [26].

Mass spectrometric conditions: A Thermo Orbitrap Exploris 120 mass spectrometer (Thermo Fisher Scientific, USA) with an electrospray ionization (ESI) source was operated in both positive- and negative-ion modes. Positive-ion mode: spray voltage, +3.50 kV; negative-ion mode: spray voltage, −2.50 kV; sheath gas: 40 arb; auxiliary gas: 10 arb; capillary temperature: 325 °C. Full-scan MS was acquired at a resolution of 60,000 (*m*/*z* 100–1000). HCD was used for MS/MS at 30% NCE and 15,000 resolution, fragmenting the top 4 precursor ions. Dynamic exclusion was applied to reduce redundant MS/MS information [27].

#### 2.8.4. Non-Targeted Metabolomics Data Processing

Raw MS files were converted to mzXML format with the MSConvert tool in ProteoWizard (v3.0.8789). Peak picking, filtering, and alignment were performed with the R package XCMS (version 3.24.1, parameters: bw = 2, ppm = 15, peakwidth = c(5,30), mzwid = 0.015, mzdiff = 0.01, method = “centWave”) to generate a quantitative feature matrix. Total-peak-area normalization was subsequently applied to correct systematic variations.

#### 2.8.5. Metabolite Identification

In the identification process, the HMDB, MassBank, LipidMaps, mzCloud, KEGG, and the in-house metabolite standard spectral library of GeneBio were searched. The exact mass and adduct ions obtained from the precursor ion in MS were used to predict the molecular formula and match against the databases. Metabolites with MS/MS spectra in the quantification list were further validated by comparing their fragment ions with those in the reference MS/MS spectra, enabling level-2 identification.

### 2.9. LC-MS/MS-Based Proteomic Profiling of RCFS

RCFS obtained from Section 2.2 was snap-frozen with liquid nitrogen and lyophilized; the resulting sample was sent to Tgene Biotech (Shanghai, China) Co., Ltd. for proteomic analysis, with sterile NB medium supernatant subjected to the same treatment as the negative control.

#### 2.9.1. Protein Extraction

An aliquot of lyophilized RCFS was resuspended in urea lysis buffer containing protease inhibitors, vortexed for 3 min, and sonicated on ice (30 Hz, 10 s on/10 s off, total 120 s). The lysate was heated at 95 °C for 5 min, cooled, and centrifuged at 12,000 rpm, 4 °C, for 20 min. Ten microliters of the supernatant was withdrawn for quantification; the remainder was stored at −80 °C until further use. The protein concentration, determined by BCA assay, was 15.9 mg/mL.

#### 2.9.2. Proteolytic Digestion and Desalting

Extracted proteins were diluted to 50 µL with urea lysis buffer, reduced and alkylated with 6 µL of the appropriate reagent, vortexed, briefly spun, and incubated at 95 °C. Trypsin was added at an enzyme-to-protein ratio of 1:50 (*w*/*w*), followed by incubation at 37 °C. Digestion was terminated by acidification, and the peptides were desalted on C18 spin columns. The eluate was collected and vacuum-dried.

#### 2.9.3. LC-MS/MS Instrument Conditions

Peptides were dissolved in 0.1% formic acid and analyzed on a VanquishNeo UHPLC system (Thermo Fisher Scientific) coupled to an Orbitrap Astral mass spectrometer (Thermo Fisher Scientific) via a homemade analytical column (15 cm × 100 µm, 1.7 µm). Mobile phases A: 98% water/2% acetonitrile/0.1% formic acid; mobile phases B: 80% acetonitrile/20% water/0.1% formic acid. Gradient (flow 0.3 µL/min): 0–1 min 8% B → 17% B; 1–5.5 min 17% B → 55% B; 5.5–7 min 55% B → 99% B; 7–8 min 99% B. Database searching was performed with DIA-NN18.1 against the Swiss-Prot database; detailed parameter settings are listed in Table 1. Statistical analyses were carried out with three replicate injections per sample. Protein identifications were filtered at a 1% protein-level false discovery rate (FDR).

### 2.10. Statistical Analysis

The results from three replicate experiments are expressed as mean ± standard deviation (SD). Statistical analysis was performed using Microsoft Excel 2016. Data were analyzed by one-way ANOVA followed by Tukey’s HSD test. Student’s *t*-test and F-test were used to compare differences between two groups and among three or more groups, respectively. In all experiments, * *p* < 0.05 was considered statistically significant.

## 3. Results

### 3.1. Ethanol PrecipitationEnrichment of AFB1-Degrading Active Constituents from RCFS

Because the specific active constituents responsible for AFB1 degradation by RCFS remain unknown, and activity is located exclusively in the cell-free supernatant, we assumed the agent(s) to be water-soluble and tentatively assigned them to proteinase, glycoprotein, or polysaccharide categories. The polar groups of proteins engage in unfavorable interactions with ethanol; raising the ethanol concentration intensifies these unfavorable contacts between hydrophilic moieties and the organic solvent, decreasing protein solubility [28]. Likewise, ethanol—less polar than water—disrupts the hydration shell formed by water molecules around hydrophilic, bioactive polysaccharides, reducing their solubility in the supernatant [29]. Ethanol precipitation requires no sophisticated equipment, is inexpensive, comparatively safe, and environmentally benign, making it suitable for large-scale industrial application. The protein contents of RCFS and RCFSC-EP were determined by the Coomassie Brilliant Blue G-250 assay, yielding 24.53 μg/mL and 20.40 μg/mL, respectively, demonstrating an 83.16% recovery of RCFS proteins by ethanol precipitation.

**Figure 1:**
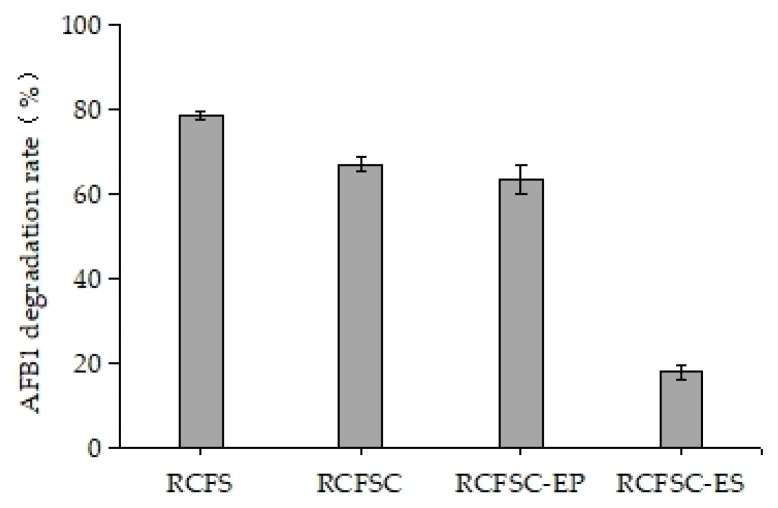
Effect of vacuum concentration and ethanol precipitation on AFB1-degrading activity of RCFS.

We therefore evaluated the feasibility of using ethanol precipitation to concentrate the AFB1-degrading constituents in RCFS; the results are shown in Figure 1. Similarly to R. erythropolis [15], Stenotrophomonas maltophilia 35-3 [23], and Pseudomonas aeruginosa N17-1 [30], RCFS prepared from R. opacus PD630 degraded AFB1 without prior substrate exposure, indicating that the activity is constitutively expressed during normal growth. Under standard assay conditions (AFB1 800 µg/L, 30 °C, 160 rpm, 24 h), RCFS removed 78.47% of AFB1. After 10-fold vacuum concentration at 55 °C, degradation by RCFSC dropped to 66.99%, revealing a significant loss of activity (*p* < 0.05). We hypothesize that the AFB1-degrading component(s) may possess a cavity-containing tertiary structure that could undergo irreversible alteration under reduced pressure; however, this remains to be verified by biophysical methods such as CD spectroscopy or enzyme assays. Ethanol precipitation retained 80.66% of activity in the pellet (RCFSC-EP), while the supernatant (RCFSC-ES) showed only 18.01% activity, confirming effective enrichment. Activity in both fractions implies multiple degradation pathways and diverse active components. Ethanol precipitation simultaneously recovered >80% of both AFB1-degrading activity and total protein from RCFS, suggesting a certain correlation between protein content and AFB1 degradation.

### 3.2. Effect of Temperature on AFB1-Degrading Activity of RCFSC-EP

**Figure 2:**
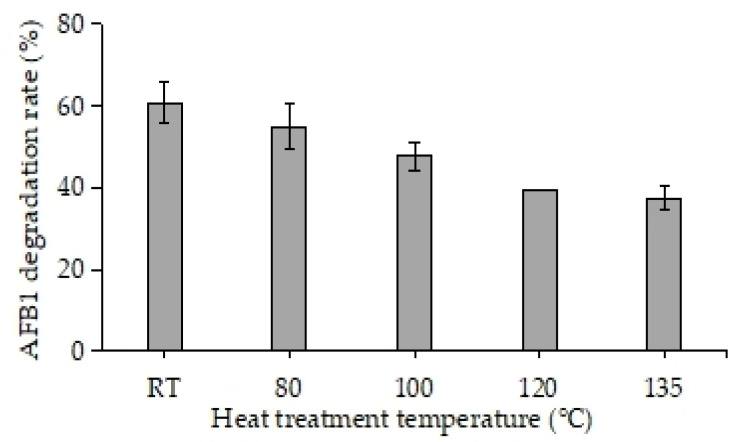
Effect of temperature on AFB1-degrading activity of RCFSC-EP (RT: room temperature).

The influence of temperature on the degradation of AFB1 by RCFSC-EP is illustrated in Figure 2. After treatment at 80 °C for 20 min, the AFB1-degrading ability was unaffected, exhibiting no significant difference (*p* > 0.05) compared with the RT control (mean ± SD: 60.89 ± 4.89% vs. 55.00 ± 5.42%; two-tailed Student’s *t*-test, *p* = 0.17). However, as the heat treatment temperature gradually increased (≥100 °C), the ability of RCFSC-EP to degrade AFB1 showed a downward trend, indicating that the active components capable of degrading AFB1 were affected by heat treatment. Notably, after high-temperature treatment at 135 °C, the degradation rate of RCFSC-EP on AFB1 was still up to 37.47%, indicating that some active components have excellent heat resistance. It is hypothesized that there may be multiple AFB1 degradation active components in RCFSC-EP, some of which can withstand higher heat treatment temperatures and still maintain the ability to degrade AFB1, possessing excellent thermal stability. This thermal stability, which can withstand up to 135 °C, indicates that RCFSC-EP can adapt to complex and diverse degradation environments and can serve as an efficient catalytic alternative, making it one of the excellent potential applications for AFB1 degradation.

Similarly, Wang et al. [31] reported that the metabolites of *Fusarium* sp. WCQ3361 exhibited high thermal stability, retaining 99.40% of their AFB1-degrading activity even after boiling for 10 min. Shu et al. [21] found that autoclaving the supernatant of *Bacillus velezensis* DY3108 at 121 °C for 30 min did not abolish AFB1-degrading activity; instead, an enhancement of degradative capacity was observed.

### 3.3. Effects of Proteinase K and EDTA-2Na on AFB1-Degrading Activity of RCFSC-EP

**Figure 3:**
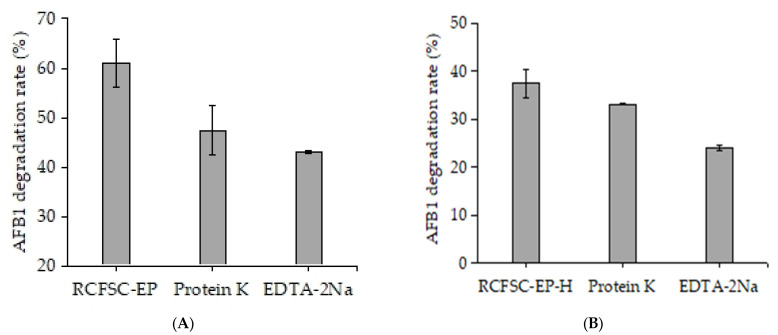
Effects of proteinase K and EDTA-2Na on AFB1-degrading activity of RCFSC-EP or RCFSC-EP-H. (**A**) RCFSC-EP; (**B**) RCFSC-EP-H: RCFSC-EP pre-heated at 135 °C for 20 min.

Detection data (Figure 3) show that after treatment with 1 mg/mL of proteinase K, the ability of RCFSC-EP to degrade AFB1 significantly decreased; a similar trend was also observed in the RCFSC-EP-H sample; this suggests that the process of RCFSC-EP degrading AFB1 may involve the action of proteins or enzymes. Additionally, the activity of RCFSC-EP in degrading AFB1 was inhibited by EDTA-2Na: under the presence of 10 mmol/L of EDTA-2Na, the degradation rate of RCFSC-EP on AFB1 dropped to 70.7% of the initial value (Figure 3A); heat-treated RCFSC-EP samples showed a similar trend: under the presence of the inhibitor EDTA-2Na, the degradation rate of RCFSC-EP-H on AFB1 decreased from 37.47% to 23.95% (Figure 3B). It is speculated that the active components of RCFSC-EP that degrade AFB1 may be enzyme activity centers dependent on metal ions and enzymatic characteristics.

Salihi et al. [32] also observed similar results, reporting that proteases from *Aspergillus* under 5 mmol/L of ethylenediaminetetraacetic acid (EDTA) had their activity reduced to 60% of the initial value. Sun et al. [22] isolated extracellular proteases secreted by *Lactobacillus curvatus* R5 from Harbin dry sausage, which could also be inhibited by EDTA. Similar results were also found in other bacteria capable of degrading AFB1, such as *Bacillus velezensis* [21] and *Stenotrophomonas maltophilia* 35-3 [23]; the culture supernatant treated with proteinase K showed a lower level of AFB1 degradation activity; thus, it is inferred that the active components in RCFSC-EP that degrade AFB1 may be proteases with metal ion-dependent enzyme activity centers.

### 3.4. Effect of Substrate on AFB1-Degrading Activity of RCFSC-EP

**Figure 4:**
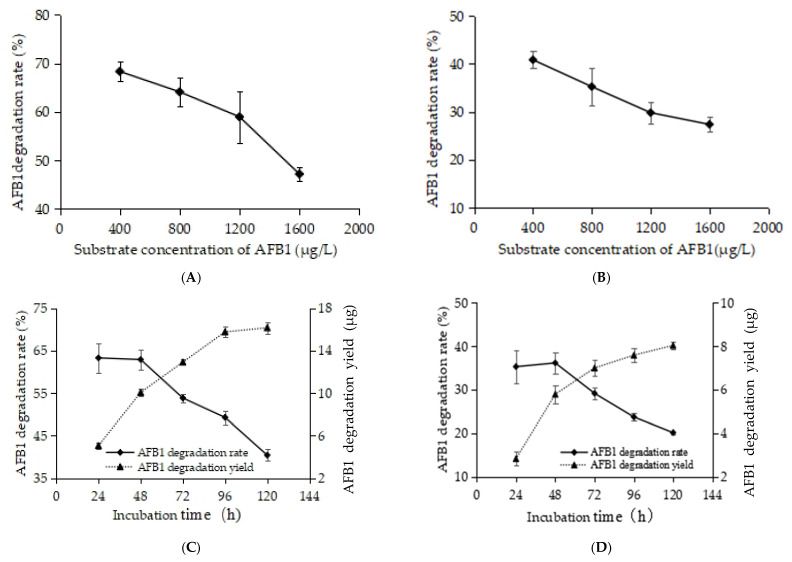
Effects of substrate concentration on AFB1 degradation by RCFSC-EP (**A**) or RCFSC-EP-H (**B**). Dynamic substrate replenishment method was employed to evaluate persistence of AFB1 degradation by RCFSC-EP (**C**) or RCFSC-EP-H (**D**).

With increasing substrate (AFB1) concentration, the degradation efficiency of RCFSC-EP toward AFB1 first remained constant and then declined, as shown in Figure 4A. When the AFB1 concentration was ≤800 μg/L, the degradation rate stayed stable. Upon further raising the substrate concentration to 1600 μg/L, the degradation rate dropped to 47.17% after 24 h incubation at 30 °C and 160 rpm, presumably because the enzyme-active sites became fully saturated at the excessive substrate level, leading to substrate inhibition. RCFSC-EP samples subjected to heat treatment at 135 °C for 20 min exhibited a similar substrate-inhibition profile (Figure 4B), indicating that the active component(s) responsible for AFB1 degradation also possess high thermal stability.

Using a dynamic substrate replenishment method, 8 μg of AFB1 was added every 24 h to the RCFSC-EP reaction mixture to assess the persistence of the degradative activity. As shown in Figure 4C, the degradation rate declined progressively from 63.29% to 40.40% with continuous AFB1 supplementation; nevertheless, the cumulative amount of AFB1 degraded kept increasing, indicating that the active principles remained functional for at least 5 d at 30 °C, conferring practical value. Heat-treated RCFSC-EP-H exhibited essentially the same persistence profile, further confirming the thermotolerance of the active components (Figure 4D). Similarly, the cell-free supernatant of *Pseudomonas aeruginosa* N17-1 displayed enhanced AFB1 detoxification with prolonged incubation, with its protein(ase) remaining stable for one week at 37 °C [30]. The exceptional thermostability and prolonged retention of AFB1-degrading activity exhibited by RCFSC-EP underscore its considerable potential for practical applications.

### 3.5. Characterization of AFB1 Degradation Products by RCFS

Compared with genomics, transcriptomics, and proteomics, metabonomics offers superior discriminatory power, higher throughput, and lower cost, and has become a key component of systems biology. The usual strategies are targeted analysis and metabolic profiling (untargeted analysis); the latter provides a global overview of the metabolome, enabling the detection of thousands of metabolites in the absence of prior information [26], thereby offering new insights into organism-wide metabolism.

#### 3.5.1. Identification of AFB1 Degradation Products Formed by RCFS

Metabolites in RCFS-AFB1 and RCFS-H-AFB1 were identified against RCFS-CK. Exact mass and adducts from MS provided molecular weights and formulas, which were matched to databases; MS/MS spectra were further compared for fragment ions to achieve level-2 identification. RCFS-AFB1 yielded six products: C_17_H_12_O_7_, C_17_H_12_O_8_, C_9_H_10_O_4_, C_17_H_14_O_7_, C_16_H_14_O_5_, and C_11_H_10_O_5_. RCFS-H-AFB1 generated only four of these, lacking C_17_H_14_O_7_ and C_16_H_14_O_5_. Degradation products and their confidence levels for identification under the Metabolomics Standards Initiative (MSI) are listed in Table 2.

#### 3.5.2. Identification and Profiling of AFB1 Degradation Products by RCFS

**Figure 5:**
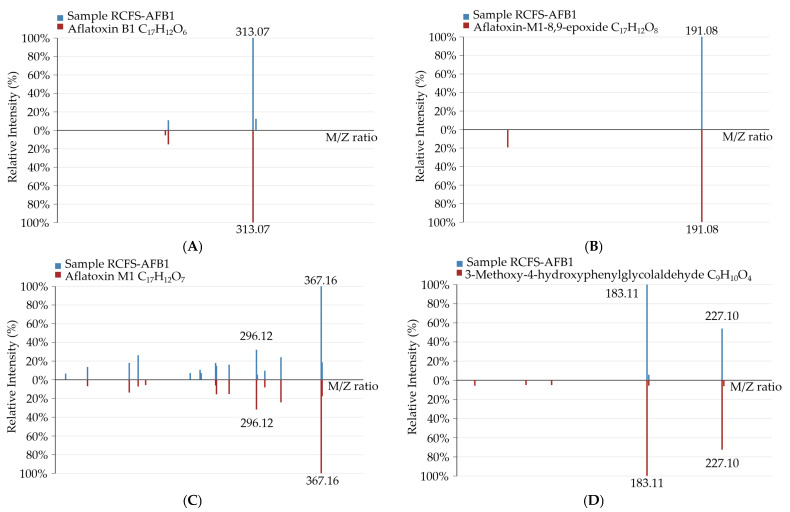
The four novel compounds in the RCFS-AFB1-treated group were unambiguously identified as AFB1 (**A**); aflatoxin-M1-8,9-epoxide (**B**); aflatoxin M1 (**C**); and 3-methoxy-4-hydroxyphenylglycolaldehyde (**D**).

Compared with RCFS-CK, new molecular-ion peaks appeared in the RCFS-AFB1-treated metabolomic chromatograms at *m*/*z* 313.0699 ([M + H]^+^), 327.0511 ([M − H_2_O + H]^+^), 367.1544 ([M + K]^+^), and 227.0533 ([M + HCOO]^−^), corresponding to molecular formulas C_17_H_12_O_6_, C_17_H_12_O_8_, C_17_H_12_O_7_, and C_9_H_10_O_4_. Matching exact masses, adducts, and MS/MS fragment ions against databases enabled level-2 identification as AFB1, aflatoxin-M1-8,9-epoxide, aflatoxin M1, and 3-methoxy-4-hydroxyphenylglycolaldehyde. The identification spectra are shown in Figure 5.

**Figure 6:**
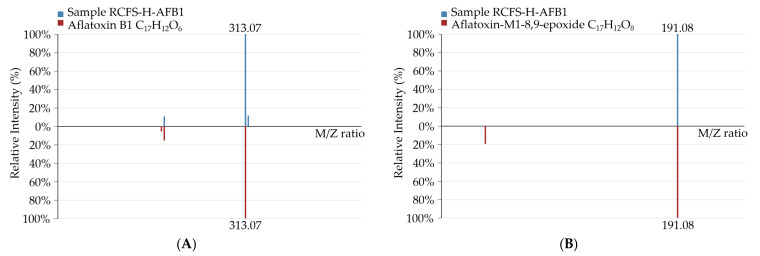

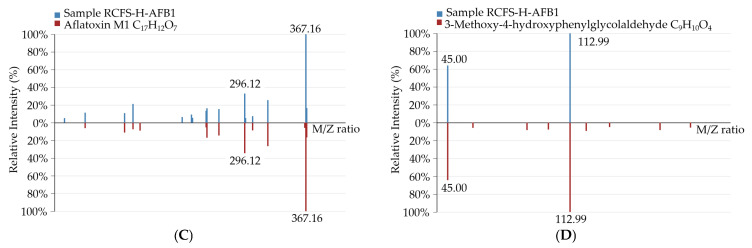
The four novel compounds in the RCFS-H-AFB1-treated group were unambiguously identified as AFB1 (**A**); aflatoxin-M1-8,9-epoxide (**B**); aflatoxin M1 (**C**); and 3-methoxy-4-hydroxyphenylglycolaldehyde (**D**).

Compared with RCFS-CK, new molecular-ion peaks emerged in the RCFS-H-AFB1 metabolomic chromatogram at *m*/*z* 313.0686 ([M+H]^+^), 327.0511 ([M-H_2_O+H]^+^), 367.1581 ([M+K]^+^), and 181.0499 ([M-H]^−^), corresponding to the formulas C_17_H_12_O_6_, C_17_H_12_O_8_, C_17_H_12_O_7_, and C_9_H_10_O_4_, respectively. Matching exact masses, adducts, and MS/MS fragment ions against databases enabled level-2 identification as AFB1, aflatoxin-M1-8,9-epoxide, aflatoxin M1, and 3-methoxy-4-hydroxyphenylglycolaldehyde; the identification spectra are presented in Figure 6.

Additionally, the RCFS-AFB1 metabolomic MS revealed molecular-ion peaks at *m*/*z* 313.0698 ([M − H_2_O + H]^+^), 285.0772 ([M − H]^−^), and 223.0618 ([M + H]^+^), corresponding to formulas C_17_H_14_O_7_, C_16_H_14_O_5_, and C_11_H_10_O_5_ (330.74, 286.08, and 222.05 Da, respectively).

In RCFS-H-AFB1, a peak at *m*/*z* 223.0624 ([M + H]^+^, C_11_H_10_O_5_, 222.05 Da) was detected.

None of these ions were present in RCFS-CK, implying that they are degradation products formed during the incubation of AFB_1_ with RCFS or RCFS-H.

#### 3.5.3. Proposed AFB1 Degradation Pathway by RCFS

**Figure 7:**
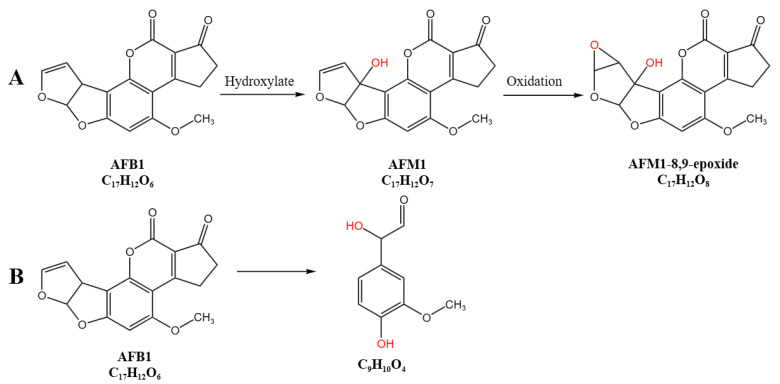
The possible degradation pathways of AFB1 By RCFS. (**A**) AFB1 is first hydroxylated by RCFS to form AFM1; subsequently, AFM1 is epoxidized to yield AFM1-8,9-epoxide; (**B**) the bisfuran and coumarin structures of AFB1 may both be destroyed by RCFS, degrading into the product C_9_H_10_O_4_.

By analyzing the metabolomic data of the samples, the treated groups RCFS-AFB1 and RCFS-H-AFB1 both revealed the degradation products AFM1 and AFM1-8,9-epoxide. AFM1 bears a hydroxyl group in its spatial molecular structure, enabling conjugation with glucuronic acid and resulting in low toxicity-only about 10% of the mutagenicity exhibited by AFB1 [33,34]. We therefore propose that one RCFS-mediated degradation route for AFB1 proceeds as follows: AFB1 is first hydroxylated by RCFS to form AFM1, which is subsequently epoxidized to AFM1-8,9-epoxide (pathway illustrated in Figure 7A); this route displays remarkable thermostability. The epoxide may next react with glutathione (GSH) to yield the putative conjugate AFM1-8,9-GSH; however, no such metabolite was detected in our metabolomic analyses.

The detection of the metabolite C_9_H_10_O_4_ (3-methoxy-4-hydroxyphenylglyoxal) led us to postulate an additional RCFS-catalyzed AFB1 degradation pathway, depicted in Figure 7B, which likewise exhibits high thermotolerance. The molecular structure of C_9_H_10_O_4_ indicates that both the bis-furan and coumarin moieties of AFB1 are cleaved, corroborating the detoxifying capacity of RCFS; however, the detailed reaction sequence and underlying mechanism remain unclear. Additionally, HPLC chromatograms confirmed that the metabolite generated upon AFB1 depletion is structurally distinct from the parent compound and remains undetectable at its characteristic retention time. Similarly, Zhou et al. [35] reported that horseradish peroxidase (HRP)-mediated degradation of AFB1 yields a product of *m*/*z* 183.0729 ([M + H]^+^, C_9_H_10_O_4_), and proposed that the lactone and bis-furan rings of AFB1 are disrupted during the process.

**Figure 8:**
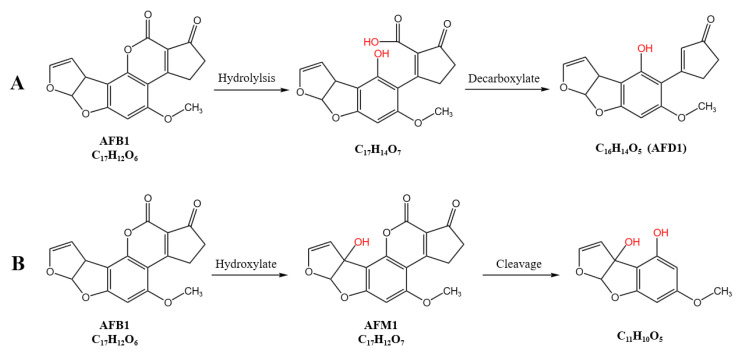
The possible degradation pathways of AFB1 By RCFS. (**A**) RCFS catalyzes AFB1 to form a β-keto-acid structure, followed by lactone ring hydrolysis and decarboxylation of the opened lactone to yield AFD1. (**B**) After AFB1 is hydroxylated to AFM1 by RCFS, it may undergo lactone hydrolysis, decarboxylation, and cleavage to form C_11_H_10_O_5_, retaining the bisfuran moiety but losing the characteristic lactone carbonyl and cyclopentenone ring of AFB1.

Additionally, in the RCFS-AFB1 treatment group, the metabolites at *m*/*z* 330.74 (C_17_H_14_O_7_), 286.08 (C_16_H_14_O_5_), and 222.05 (C_11_H_10_O_5_) were inferred to be degradation products generated during RCFS incubation with AFB1. As previously reported by Liu et al. [19], co-culture of AFB1 with *R. opacus* PD630 in NB medium altered the coumarin structure and destroyed the lactone ring, as determined by TLC analysis. We propose that active component(s) in RCFS catalyze formation of a β-keto-acid intermediate. Subsequent hydrolysis of the lactone ring followed by decarboxylation of the opened lactone yields the compound at *m*/*z* 285.08, designated AFD1, in which the characteristic lactone carbonyl of AFB1 is lost; the putative pathway is detailed in Figure 8A. Likewise, Ludmila et al. [14] reported that *R. erythropolis* secretes multiple enzymes acting on aromatic compounds and, using high-resolution FTMS, detected lactone ring cleavage to a β-keto-acid that undergoes decarboxylation to yield C_16_H_14_O_5_ (AFD1). The formation of the C_11_H_10_O_5_ metabolite is postulated to proceed via hydroxylation of AFB1 to AFM_1_, followed by lactone hydrolysis, decarboxylation, and fragmentation, retaining the bis-furan moiety but eliminating both the lactone carbonyl and the cyclopentenone ring of AFB1; the proposed sequence is illustrated in Figure 8B. In the RCFS-H-AFB1 group (135 °C, 20 min), only the C_11_H_10_O_5_ product (*m*/*z* 223.06) was detected, indicating that heat treatment may suppress the metabolic routes leading to C_17_H_14_O_7_ and C_16_H_14_O_5_.

Metabolomic profiling revealed that RCFS degrades AFB1 into six distinct products, three of which were identified as aflatoxin-M1-8,9-epoxide, aflatoxin M1, and 3-methoxy-4-hydroxyphenylglycolaldehyde. The remaining three metabolites were tentatively assigned as follows: C_17_H_14_O_7_ and C_16_H_14_O_5_ (AFD1) arise from hydrolytic opening of the AFB1 lactone ring, whereas C_11_H_10_O_5_ is proposed to originate from lactone hydrolysis and decarboxylation of AFM1 followed by cleavage of the cyclopentenone ring. Comparative analysis demonstrated that thermal treatment (135 °C, 20 min) selectively perturbs the biotransformation route leading to C_17_H_14_O_7_ and C_16_H_14_O_5_ (AFD1), while the other three putative degradation pathways remained unaffected. The metabolomic profile of the RCFS-H-AFB1 sample set thus provides compelling evidence that the AFB1-degrading principle(s) in RCFS possess exceptional thermostability (135 °C), surviving high-temperature sterilization procedures and offering a promising tool for eliminating AFB1 contamination in food and feed matrices.

### 3.6. Proteomic Profile of RCFS

Using NB blank medium as control, three biological replicates of RCFS yielded a total of 9059 peptides after enzymatic digestion and LC-MS/MS analysis, finally leading to the identification of 1136 proteins, evidencing a rich enzyme/protein repertoire in RCFS. Given that previous studies have attributed microbial AFB1 degradation mainly to enzymatic reactions classifiable as oxidative, reductive, and lactonase–hydrolytic processes [13], we subjected the identified proteins to functional annotation against the Swiss-Prot database with DIA-NN18.1. The results revealed the presence of detoxifying oxidases, reductases, and glutathione S-transferases in RCFS, whereas no lactonase was detected; details are given in Table 3.

In RCFS, we identified the glutathione S-transferases (GSTs) GTT1, GSTP1, GSTA1, GSTM1, and GSTO1. Alnasser et al. [36] defined GSTs as an enzyme superfamilythat detoxifies diverse harmful compounds via conjugation with glutathione (GSH). Mazari et al. [37] noted that GSTs display pronounced structural similarity and partial functional redundancy; their cardinal role is to protect the cell by conjugating a broad spectrum of toxic molecules to GSH. The finely tuned balance of GST-mediated detoxification is indispensable for cellular homeostasis. Studies have established that GSTs play a pivotal role in AFB1 detoxification by catalyzing the addition of reduced GSH to AFB1-8,9-epoxide (AFBO), thereby preventing DNA-adduct formation and diminishing toxicity [38]. Deng et al. [39] over-expressed five GST isoforms in chicken Leghorn male hepatoma cells and observed that GSTA2X, GSTA3, GSTT1L, GSTZ1-1, and GSTZ1-2 markedly increased AFBO–GSH formation and alleviated AFB1-induced DNA damage, with GSTA2X exhibiting the highest detoxification efficacy. We therefore hypothesize that the GSTs detected in RCFS participate in catalyzing the conjugation of glutathione to AFM1-8,9-epoxide, resulting in attenuated toxicity.

In RCFS, we also identified aldo-keto reductases (AKRs): AKR1A1 and aflatoxin B1 aldehyde reductase (AFAR) (AKR7A2, and Akr7a2). AKRs constitute an NADPH-dependent oxidoreductase superfamily that reduces carbonyls to the corresponding alcohols, converting aldehydes to primary alcohols and ketones to secondary alcohols. AKR1 is among the largest families within the superfamily and performs broad biological functions including carbonyl detoxification [40]. In experimental animal models, AFB1-8,9-epoxide undergoes rapid hydrolysis in aqueous medium to yield AFB1-dihydrodiol, which, under base catalysis, rearranges to establish an equilibrium with AFB1-dialdehyde. AFAR catalyzes the reduction in this protein-reactive AFB1-dialdehyde to the non-reactive AFB1-dialcohol [41]. AKR7A belongs to the AKR7 subfamily, phylogenetically closest to AKR1. AKR7A enzymes employ NADPH as cofactor to convert a wide range of aldehydes and ketones to alcohols, thereby either bio-activating or detoxifying carbonyl compounds; members of the AKR7A subfamily confer potent protection against chemical-induced hepatotoxicity. AKR7A2, the first human AKR7A member identified in 1998, exhibits substrate specificity toward numerous endogenous and xenobiotic carbonyls and participates in the detoxification of diverse aldehydes and ketones. In vitro studies demonstrate that AKR7A2 protects against AFB1 toxicity by reducing the protein-bound AFB1-dialdehyde to the non-bound AFB1 mono- or dialcohol AFB1 [42]. AKRs are also widespread in microorganisms; Xie et al. [43] induced the expression of the *At*AKR297 gene from *Armillariella tabescens* in *Escherichia coli* BL21(DE3) and demonstrated that the NADPH-dependent *At*AKR297 reduces to the less-toxic aflatoxicol (AFL, C_17_H_14_O_6_).

In addition to the GST and AKR families, the peroxidase family was also detected in RCFS, exemplified by the putative heme-binding peroxidase (CCPR2_USTMA) and catalase-peroxidase (KATG_RHOJR). Studies have demonstrated that peroxidases are highly effective in mycotoxin degradation. Marimon et al. [44] reported that 0.015 U/mL commercial peroxidase (POD) degraded 97% of 0.5 µg/L AFB1 under defined conditions, and POD also degraded AFB1 when added to milk or beer. Zhou et al. [35] showed that horseradish peroxidase (HRP) catalyzed AFB1 degradation in the presence of 0.1 mmol H_2_O_2_, yielding a C_9_H_10_O_4_ product in which the lactone and difuran rings of AFB1 were destroyed. Qin et al. [45] achieved the effective degradation of AFB1 to AFB1-diol by the recombinant dye-decolorizing peroxidase BsDyP from *Bacillus subtilis* SCK6 expressed in *E. coli* in the presence of Mn^2+^. Loi et al. [46] enzymatically degraded AFB1 to AFQ1 in vitro using the N246A variant of dye-decolorizing peroxidase DypB (Rh-DypB) from *R. jostii* expressed in *E. coli*, resulting in significantly reduced toxicity.

Additionally, the NADPH-dependent carbonyl reductase CBR1, belonging to the short-chain dehydrogenase/reductase (SDRs) family, was identified in RCFS. Carbonyl reductases (CBRs) exhibit broad substrate specificity toward numerous endogenous and exogenous carbonyl compounds and reduce various xenobiotic quinones derived from polycyclic aromatic hydrocarbons, participating in organismal detoxification processes [47]. Studies have shown that CBR1 metabolizes exogenous carbonyls and quinones to less-toxic products for excretion, protecting organisms from carbonyl-induced oxidative stress [48].

Proteomic profiling revealed that RCFS contains a repertoire of enzymes—including GSTs, AKRs, peroxidases, catalase-peroxidases, and CBRs—that are capable of binding, oxidizing, or reducing AFB1. The abundance of these enzymes indicates that AFB1 degradation by RCFS proceeds through multiple parallel pathways rather than a single route and is catalyzed by a concerted action of diverse enzymes. Extracellular microbial metabolism relies on a broad spectrum of enzymes to execute distinct catalytic reactions, and toxin degradation is no exception. To circumvent toxic insult, organisms have evolved dedicated enzymes for xenobiotic bio-transformation; consequently, toxin-degrading enzymes are ubiquitous across a wide range of biological systems. The enzymes and proteins identified in RCFS can be exploited for AFB1 degradation in food and animal feed.

## 4. Discussion

Our results demonstrate that, although RCFSC-EP retains AFB1-degrading activity after exposure to 135 °C, its efficacy declines markedly as the thermal treatment temperature increases. EDTA-2Na, proteinase K, and the substrate AFB1 itself all interfere with the AFB1-degrading capacity of RCFSC-EP, indicating that the active principle(s) are predominantly enzymatic or proteinaceous. Proteomic profiling further identified RCFS proteins capable of oxidizing, reducing, or conjugating AFB1, thereby providing mechanistic evidence for the chemical nature of the bioactive components responsible for AFB1 degradation. Concomitantly, the metabolomic identification of transformation products allowed us to propose a plausible AFB1 degradation pathway catalyzed by RCFS.

Limitations and future perspectives: Although four putative AFB1 degradation routes have been delineated, unambiguous pathway confirmation awaits isolation and structural elucidation of the individual intermediates. The detoxification efficacy of RCFS-treated AFB1 remains to be validated by Ames testing and/or cell-based cytotoxicity assays. Moreover, while ethanol precipitation enriches the active fraction, the key enzyme(s) or protein(s) responsible for AFB1 degradation must be purified to homogeneity and functionally verified.

## 5. Conclusions

In this study, ethanol precipitation was employed to enrich the active components responsible for AFB1 degradation by RCFS, and it was found that this method could recover the majority of the active constituents. By integrating metabolomic and proteomic analyses, we conducted an in-depth investigation into the active components, metabolites, and pathways involved in the RCFS-mediated degradation of AFB1. Based on the multi-omics integrative analysis, the degradation of AFB1 by RCFS exhibited pathway and product diversity. The key contributors were identified as proteins or enzymes, including GSTs, AKRs, peroxidases, catalase-peroxidases, and CBRs. These enzymes metabolized AFB1 into six metabolites via four putative metabolic pathways, most of which demonstrated remarkable thermostability. These findings offer novel insights and research directions for elucidating the AFB1-degrading capability of RCFS.

## Figures and Tables

**Table 1:** DIA-NN 1.8.1 software search database retrieval parameters settings.

Parameters	Value
Enzyme	Trypsin
Static modification	Carbamidomethyl/+57.021 Da (C)
Dynamic modification	Oxidation/+15.995 Da (M); Acetyl/+42.011 Da (N-Terminal)
Precursor charge range	1–4
Peptide length range	7–30
Precursor mz range	300–1800
Fragment ion mz range	200–1800
Max missed cleavages	2

**Table 2:** Products generated from AFB1 degradation by RCFS or RCFS-H.

Treatment Group	Molecular Formula	Molecular Weight	*m*/*z* Ratio	Identification Level
RCFS-AFB1 ^1^	C_17_H_12_O_7_	328.06	367.1554	Level 1
	C_17_H_12_O_8_	344.05	327.0511	Level 1
	C_9_H_10_O_4_	182.06	227.0533	Level 2
	C_17_H_14_O_7_	330.074	313.0698	Level 3
	C_16_H_14_O_5_	286.08	285.0772	Level 3
	C_11_H_10_O_5_	222.05	223.0618	Level 3
RCFS-H-AFB1 ^2^	C_17_H_12_O_7_	328.06	367.1581	Level 1
	C_17_H_12_O_8_	344.05	327.0511	Level 1
	C_9_H_10_O_4_	182.06	181.0499	Level 2
	C_11_H_10_O_5_	222.05	223.0624	Level 3

^1^ Cell-free supernatant of *R. opacus* PD630 for AFB1 degradation treatment group; ^2^ cell-free supernatant of *R. opacus* PD630 heat-treated at 135 °C for 20 min for AFB1 degradation treatment group.

**Table 3:** Oxidases, reductases, and glutathione S-transferases with putative detoxification activity identified in RCFS.

Protein IDs	Genes	Protein Description	Protein Families
P40582	*GTT1*	Glutathione S-transferase 1	GST superfamily
P80031	*GSTP1*	Glutathione S-transferase P	GST superfamily, Pi family
Q28035	*GSTA1*	Glutathione S-transferase A2	GST superfamily, Alpha family
P09488	*GSTM1*	Glutathione S-transferase Mu 2	GST superfamily, Mu family
Q9N0V4	*GSTM1*	Glutathione S-transferase 2	GST superfamily, Mu family
Q9N1F5	*GSTO1*	Glutathione S-transferase omega-1	GST superfamily, Omega family
Q3ZCJ2	*AKR1A1*	Aldo-keto reductase family 1 member A1	Aldo/keto reductase family
O43488	*AKR7A2*	Aflatoxin B1 aldehyde reductase member 2	Aldo/keto reductase family, Aldo/keto reductase 2 subfamily
Q8CG76	*Akr7a2*	Aflatoxin B1 aldehyde reductase member 2	Aldo/keto reductase family, Aldo/keto reductase 2 subfamily
Q4PD66	*CCP2*	Putative heme-binding peroxidase	Peroxidase family, Cytochrome c peroxidase subfamily
Q0S5Y0	*katG*	Catalase-peroxidase	Peroxidase family, Peroxidase/catalase subfamily
Q3SZD7	*CBR1*	Carbonyl reductase [NADPH] 1	Short-chain dehydrogenases/reductases (SDR) family

## Data Availability

The original contributions presented in this study are included in the article. Further inquiries can be directed to the corresponding author.

## Appendix A

Dichloromethane, formic acid, disodium ethylenediaminetetraacetate (EDTA-2Na), and proteinase K were purchased from Shanghai TCI Co., Ltd. (Shanghai, China). LC-MS-grade acetonitrile and methanol were purchased from Fisher Scientific (Loughborough, UK). 2-Amino-3-(2-chlorophenyl)propanoic acid and AFB1 standard were from Aladdin (Shanghai, China). Ammonium formate was from Sigma-Aldrich (Shanghai, China).

A high-speed refrigerated centrifuge was procured from Hunan Xiangyi Laboratory Instrument Development Co., Ltd. (Changsha, China). A vortex mixer was supplied by Haimen Kylin-Bell Lab Instruments Co., Ltd. (Haimen, China). An ultrasonic cleaner was obtained from Kunshan Shumei Laboratory Equipment Co., Ltd. (Kunshan, China).

A tissue grinder was purchased from Zhejiang Meibi Experimental Instrument Co., Ltd. (Hangzhou, China). The 0.22 µm microporous membranes were provided by Tianjin Jinteng Experimental Equipment Co., Ltd. (Tianjin, China).
